# Fatty acid binding proteins are novel modulators of synaptic epoxyeicosatrienoic acid signaling in the brain

**DOI:** 10.1038/s41598-023-42504-4

**Published:** 2023-09-14

**Authors:** Sherrye T. Glaser, Kalani Jayanetti, Saida Oubraim, Andrew Hillowe, Elena Frank, Jason Jong, Liqun Wang, Hehe Wang, Iwao Ojima, Samir Haj-Dahmane, Martin Kaczocha

**Affiliations:** 1 Department of Anesthesiology, Renaissance School of Medicine, Stony Brook University, Stony Brook, NY USA; 2 Department of Biological Sciences, Kingsborough Community College, Brooklyn, NY USA; 3 Department of Chemistry, Stony Brook University, Stony Brook, NY USA; 4 Department of Pharmacology and Toxicology, Jacobs School of Medicine and Biomedical Sciences, University at Buffalo, State University of New York, Buffalo, NY USA; 5 Institute of Chemical Biology and Drug Discovery, Stony Brook University, Stony Brook, NY USA; 6 Stony Brook University Pain and Analgesia Research Center (SPARC), Renaissance School of Medicine, Stony Brook University, Stony Brook, NY USA

**Keywords:** Lipids, Molecular modelling, Synaptic transmission, Transporters in the nervous system

## Abstract

Fatty acid binding proteins (FABPs) govern intracellular lipid transport to cytosolic organelles and nuclear receptors. More recently, FABP5 has emerged as a key regulator of synaptic endocannabinoid signaling, suggesting that FABPs may broadly regulate the signaling of neuroactive lipids in the brain. Herein, we demonstrate that brain-expressed FABPs (FABP3, FABP5, and FABP7) interact with epoxyeicosatrienoic acids (EETs) and the peroxisome proliferator-activated receptor gamma agonist 15-deoxy-Δ^12,14^-Prostaglandin J2 (15d-PGJ_2_). Among these lipids, EETs displayed highest affinities for FABP3 and FABP5, and 11,12-EET was identified as the preferred FABP ligand. Similarly, 15d-PGJ_2_ interacted with FABP3 and FABP5 while binding to FABP7 was markedly lower. Molecular modeling revealed unique binding interactions of the ligands within the FABP binding pockets and highlighted major contributions of van der Waals clashes and acyl chain solvent exposure in dictating FABP affinity and specificity. Functional studies demonstrated that endogenous EETs gate the strength of CA1 hippocampal glutamate synapses and that this function was impaired following FABP inhibition. As such, the present study reveals that FABPs control EET-mediated synaptic gating, thereby expanding the functional roles of this protein family in regulating neuronal lipid signaling.

## Introduction

Fatty acid binding proteins (FABPs) are a family of intracellular chaperones that transport lipid cargoes to organelles and nuclear receptors, thereby regulating diverse processes in the brain and peripheral tissues^1, 2^. The mammalian brain expresses three FABPs: FABP3, FABP5, and FABP7, which display distinct regional and cellular expression patterns^3^, and hence physiological functions. For instance, FABP3 localizes exclusively to neurons and regulates dopamine and glutamate signaling, and is implicated in stress homeostasis^4,5^. FABP5, which is expressed by neurons and astrocytes, regulates endocannabinoid (eCB) signaling, addiction, and anxiety-related behaviors^6–9^. The distribution of FABP7 is restricted to astrocytes wherein it is involved in modulating glutamatergic signaling and sleep–wake cycle^10,11^. These findings indicate that FABPs exhibit non-overlapping cellular distribution patterns within the brain and modulate an array of neurological functions.

It is well recognized that FABPs interact with free fatty acids of varying acyl chain length^12,13^ and can likewise accommodate distinct fatty acid-derived bioactive lipids. For example, lipoxygenase products of arachidonic acid (AA) including 15-hydroxyeicosatetraenoic acid and leukotriene A4 interact with FABP5^14,15^. In addition, we previously identified the AA-derived eCBs, anandamide (AEA) and 2-arachidonoylglycerol (2-AG), as FABP ligands^7,9,16,17^. Importantly, we demonstrated that FABP5 exerts a key role in controlling eCB levels and synaptic signaling^7,9^. In contrast, prostaglandin E_2_ (PGE_2_), a cyclooxygenase product of AA does not display appreciable affinity for FABP5 and FABP7^14,15^. Collectively, these findings suggest that FABPs may bind to and regulate the signaling capacity of a subset of AA-derived lipids in the brain.

The epoxyeicosatrienoic acids (EETs) 5,6-EET, 8,9-EET, 11,12-EET, and 14,15-EET are a class of cytochrome P450-catalyzed AA metabolites that regulate diverse biological processes^18,19^. For instance, within the brain, enhancement of EET levels via inhibition of the EET inactivating enzyme soluble epoxide hydrolase (sEH) or administration of 14,15-EET dampens Alzheimer’s disease progression in a preclinical model^20,21^. At the cellular level, several EETs have been demonstrated to modulate synaptic transmission, ion channel function, and neuronal excitability in the hippocampus^22–24^. The recent findings that FABP5 controls the trafficking and signaling of eCBs^7,9^ combined with the observation that FABP3 binds to EETs^25^, highlight the possibility that these proteins may broadly regulate the signaling capacity of other AA-derived neuroactive lipids including EETs.

Similar to AA, 2-AG and AEA are substrates for cyclooxygenase-2, yielding prostaglandin glyceryl esters and prostamides, respectively^26,27^. Interestingly, while activation of CB1 receptors by 2-AG inhibits synaptic transmission, the cyclooxygenase-2 metabolite of 2-AG, prostaglandin E_2_ glyceryl ester (PGE_2_-GE), enhances excitatory glutamatergic transmission^28^. Although little is known about prostamide function in the brain, prostamide F2α (PGF_2α_-EA) modulates pain in the peripheral nervous system^29^. While FABP5 modulates AEA and 2-AG signaling in the brain, it is currently not known whether this function extends to cyclooxygenase metabolites of eCBs. Consequently, in the current study we characterized the potential interactions between all brain-expressed FABPs and various AA-derived lipids including EETs and cyclooxygenase eCB metabolites. Combining in vitro binding analyses, molecular modeling, and ex vivo electrophysiological approaches, our findings demonstrate that FABPs exert a key role in modulating synaptic EET signaling.

## Results

### Binding of neuroactive lipids to brain FABPs

**Figure 1:**
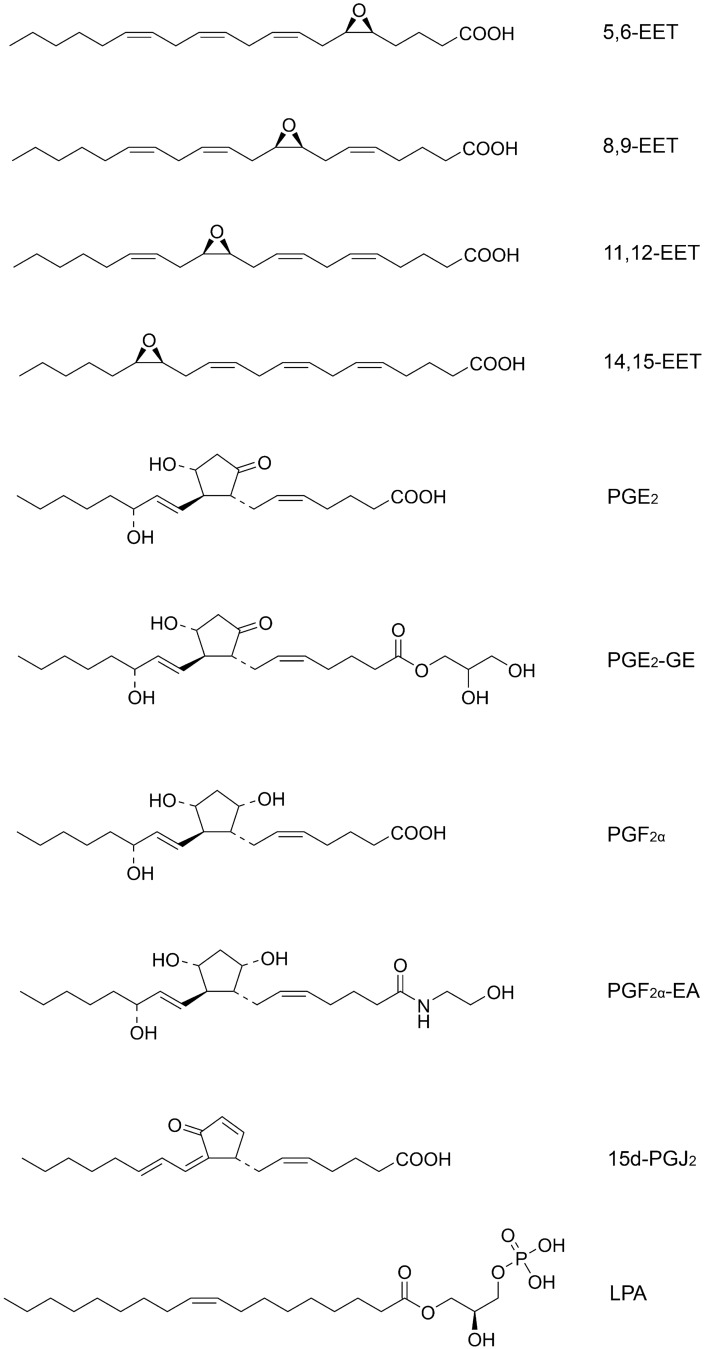
Structures of the ligands examined in this study.

**Figure 2:**
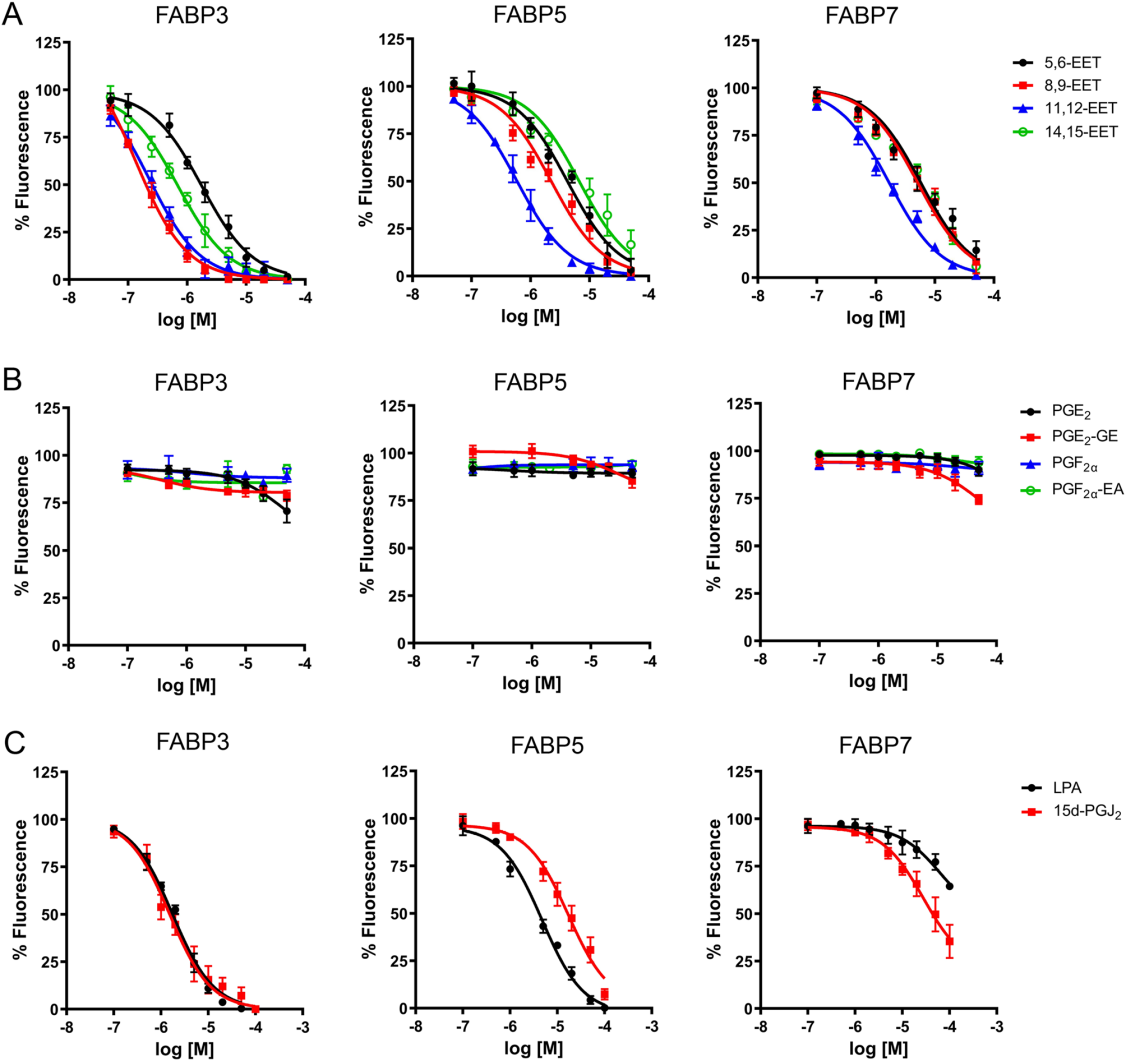
Ligand affinities for FABP3, FABP5, and FABP7. (**A**) Binding of 5,6-EET, 8,9-EET, 11,12-EET, and 14,15-EET to FABP3, FABP5, and FABP7. (**B**) Binding of PGE_2_-GE, PGE_2_, PGF_2α_-EA, and PGF_2α_ to FABP3, FABP5, and FABP7. (**C**) Binding of 15d-PGJ_2_ and LPA to the FABPs. The displacement of the fluorescent probe from each FABP is shown. Data represent mean ± S.E. (n = 4).

We first examined the binding of EETs (Fig. 1) to purified FABP3, FABP5, and FABP7 via fluorescent probe displacement assays as previously described^30,31^. 5,6-EET, 8,9-EET, 11,12-EET, and 14,15-EET bound to all three FABPs with distinct selectivity. Overall, EETs displayed highest affinities for FABP3 and the lowest for FABP7 (Fig. 2A, Table 1). For FABP3, 8,9-EET and 11,12-EET exhibited the highest affinities followed by 14,15-EET and 5,6-EET (Fig. 2A, Table 1). Similarly, 11,12-EET emerged as the preferred ligand for FABP5 followed by 8,9-EET, 5,6-EET, and 14,15-EET (Fig. 2A, Table 1). For FABP7, 11,12-EET bound with highest affinity while interactions of the remaining EETs were considerably weaker. Collectively, these results indicate that EETs bind to all three brain-expressed FABPs albeit with notable differences in specificities, with 11,12-EET emerging as the preferred ligand for FABP3, FABP5, and FABP7.

**Table 1:** Binding affinities of each ligand for FABP3, FABP5, and FABP7.

Ligand	K_i_ FABP3 (μM)	K_i_ FABP5 (μM)	K_i_ FABP7 (μM)	ClogP
5,6-EET	1.44 ± 0.12	3.57 ± 0.28	4.30 ± 0.29	5.83
8,9-EET	0.17 ± 0.02	1.87 ± 0.21	3.16 ± 0.30	5.83
11,12-EET	0.20 ± 0.03	0.52 ± 0.07	1.13 ± 0.11	5.83
14,15-EET	0.65 ± 0.08	5.56 ± 0.48	3.74 ± 0.12	5.83
PGE_2_-GE	> 50	> 50	> 50	0.91
PGE_2_	> 50	> 50	> 50	2.01
PGF_2α_-EA	> 50	> 50	> 50	1.06
PGF_2α_	> 50	> 50	> 50	2.27
15d-PGJ_2_	1.22 ± 0.14	12.29 ± 1.54	29.92 ± 5.64	5.39
LPA	1.37 ± 0.12	3.20 ± 0.19	> 50	6.29

Affinities are presented in μM ± S.E (n = 4). ClogP values were determined using ChemDraw.

We next characterized the binding of PGE_2_-GE and PGF_2α_-EA as well as their respective prostaglandin congeners, PGE_2_ and PGF_2α_. In agreement with previous work^14,15^, PGE_2_ did not interact with any of the FABPs examined (Fig. 2B, Table 1). Moreover, PGF_2α_ exhibited negligible affinity for the FABPs, and similar profiles were observed for PGE_2_-GE and PGF_2α_-EA (Fig. 2B, Table 1), indicating that these cyclooxygenase metabolites of AA, AEA, and 2-AG do not interact with brain-expressed FABPs. Previous findings indicate that the affinity of fatty acids for FABPs is inversely correlated with their aqueous solubility^32,33^. Consequently, the low affinities of these ligands may stem from their greater aqueous solubility as evidenced by low cLogP values (Table 1).

Recent evidence implicates nuclear peroxisome proliferator-activated receptor gamma (PPARγ) in the modulation of synaptic signaling^34^. FABP3 was previously shown to bind and transport oleoyl lysophosphatidic acid (LPA) to PPARγ receptors^35^, prompting us to assess its interactions with other brain expressed FABPs. We also examined whether the AA-derived PPARγ agonist 15-deoxy-Δ^12,14^-Prostaglandin J2 (15d-PGJ_2_) serves as an FABP ligand. Our results confirmed that LPA binds to FABP3 and further demonstrate that it displays a relatively comparable affinity for FABP5 while it is a poor ligand for FABP7 (Fig. 2C, Table 1). In addition, we identified 15d-PGJ_2_ as a novel FABP ligand that exhibits highest affinity for FABP3, followed by FABP5 and then FABP7 (Fig. 2C, Table 1).

### Molecular modeling of FABP-ligand interactions

We employed molecular docking to gain structural insights into FABP-lipid interactions that may confer ligand selectivity. The analysis was performed using Dock6.9 with the co-crystal structures of FABP5 with linoleic acid (PDB: 4LKT), FABP3 and FABP7 with oleic acid (PDB: 1HMS and 1FE3, respectively) serving as templates (Supplementary Fig. 1). Overall, the molecular docking analysis revealed that EETs exhibit favorable binding interactions with all three FABPs, while LPA and 15d-PGJ_2_ display enhanced binding to FABP3 and FABP5 compared to FABP7 (Table 2), consistent with the experimentally determined affinities.

**Table 2:** Grid scores of the ligands with FABP3, FABP5, and FABP7 were determined using Dock 6.9.

Ligand	FABP3 grid score (kcal/mol)	FABP5 grid score (kcal/mol)	FABP7 grid score (kcal/mol)
5,6-EET	− 53.37	− 51.27	− 49.22
8,9-EET	− 55.37	− 53.18	− 50.29
11,12-EET	− 56.66	− 55.77	− 52.59
14,15-EET	− 56.06	− 49.67	− 49.98
15d-PGJ_2_	− 53.06	− 45.35	− 40.16
LPA	− 52.89	− 49.13	− 27.75

**Figure 3:**
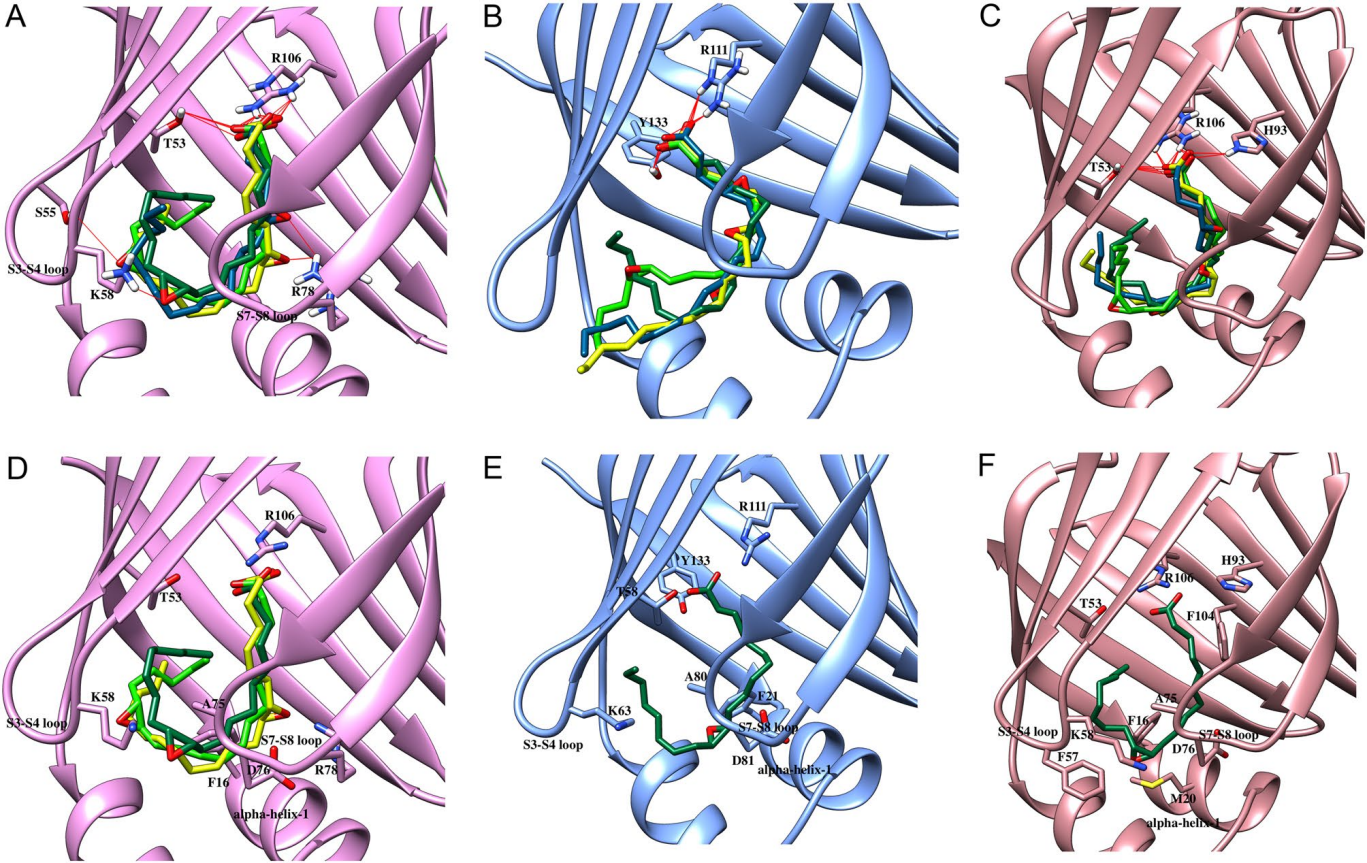
Docking of EETs in FABP3, FABP5, and FABP7. Docking poses of 5,6-EET (blue), 8,9-EET (yellow), 11,12-EET (dark green), and 14,15-EET (light green) at the binding site of (**A**, **D**) FABP3, (**B**, **E**) FABP5, and (**C**, **F**) FABP7. (**A**–**C**) Carboxylate moieties of EETs make canonical interactions with (**A**) T53 and R106 in FABP3, (**B**) R111 and Y133 in FABP5, and (**C**) T53, H93, and R106in FABP7. The epoxide groups engage in H-bonding with residues lining the binding pocket of (**A**) FABP3 to increase binding affinity. **(D**–**F)** Van der Waals interactions of EETs with FABPs. The orientation of (**D**) 8,9-EET, 11,12-EET, and 14,15-EET in FABP3, (**E**) 11,12-EET in FABP5, and (**F**) 11,12-EET in FABP7 promotes favorable van der Waals interactions to stabilize the ligands in the binding pocket.

**Figure 4:**
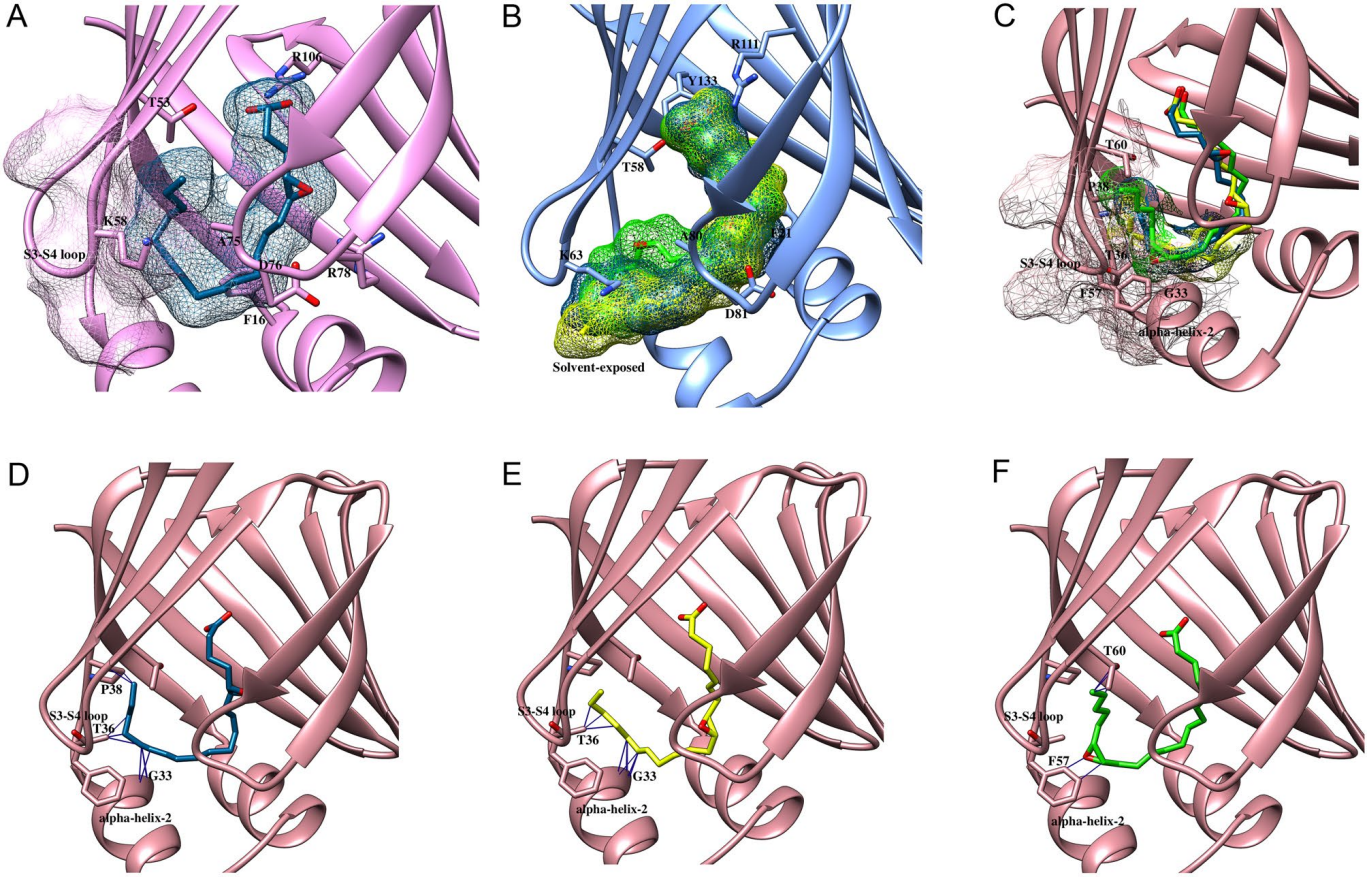
Van der Waals clashes and solvent exposure of EETs. The compact binding pocket of (**A**) FABP3 induces van der Waals clashes with 5,6-EET while that of (**C**) FABP7 results in clashes with 5,6-EET, 8,9-EET, and 14,15-EET. The wider pocket of (**B**) FABP5 leads to solvent exposure of the acyl chains of 5,6-EET, 8,9-EET, and 14,15-EET to reduce affinity. (**D**–**F**) Van der Waals clashes of (**D**) 5,6-EET with P38, T36 and G33, (**E**) 8,9-EET with T36 and G33, and (**F**) 14,15-EET with F57 and T60 in the binding pocket of FABP7.

Focusing first on the EETs, our results demonstrate that the carboxylate moiety of EETs engages in canonical interactions with R106 and T53 in FABP3, R111 and Y133 in FABP5, and R106, H93, and T53 in FABP7 (Fig. 3A–C), which position the lipids inside the binding pockets. Since the binding pockets of FABP3 and FABP7 are considerably more compact than that of FABP5 (Supplementary Fig. 2), EETs orient their acyl chains inside the FABP3 and FABP7 pockets while a more extended conformation is observed within FABP5. Due to its compact binding pocket, the epoxide group of EETs engages in H-bonding interactions with residues in FABP3 by acting as an H-bond acceptor (Fig. 3A). This orientation increases van der Waals contacts to enhance binding affinities (Fig. 3D). However, the position of the epoxide group of 5,6-EET orients the tail of the acyl chain closer to the S3-S4 loop of FABP3 (Fig. 4A), leading to van der Waals clashes with K58 that destabilize the ligand and reduce binding to FABP3.

In contrast to FABP3, 5,6-EET, 8,9-EET, and 14,15-EET attain an extended conformation within the FABP5 pocket, placing their acyl chains outside of the pocket and partially solvent exposed (Fig. 4B), which may account for the reduced binding affinity of these ligands with FABP5 (Table 1). However, the epoxide moiety of 11,12-EET is well positioned within the binding pocket and the acyl chain is stabilized through van der Waals interactions (Fig. 3E). These differential binding geometries may account for the higher affinity of 11,12-EET compared to the other ligands (Table 1, Table 2).

Among the FABPs, EETs bind to FABP7 with lowest affinities (Table 1). Molecular docking revealed that 5,6-EET, 8,9-EET, and 14,15-EET are not well accommodated within the FABP7 pocket and induce van der Waals clashes with the inward facing F57 residue in the S3-S4 loop (Fig. 4C–F, Supplementary Fig. 3), offsetting the stabilization obtained through H-bonding and reducing the affinity towards FABP7. In contrast, the orientation of the epoxide of 11,12-EET enables the formation of van der Waals interactions to stabilize the ligand within the FABP7 pocket (Fig. 3F), which may account for its higher affinity compared to the other EETs.

**Figure 5:**
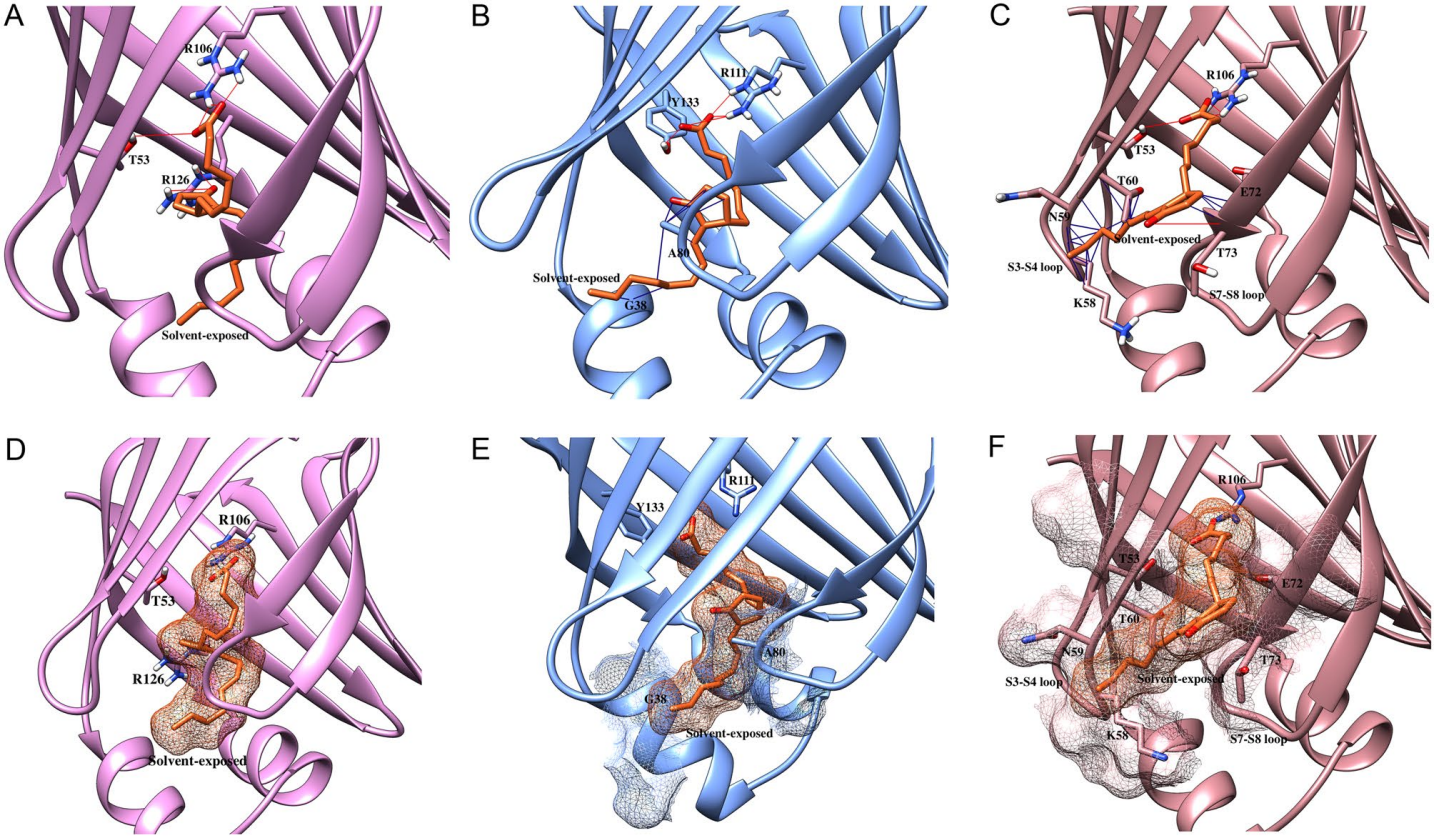
Docking poses of 15d-PGJ_2_ in FABP3, FABP5, and FABP7. (**A-C**) 15d-PGJ_2_ engages in H-bonding with (**A**) R106, T53, R126 in FABP3, (**B**) R111 and Y133 in FABP5, and (**C**) R106, T53, and T73 in FABP7. However, the rigidity of the ligand leads to exposure of the acyl chain to the solvent and induces pronounced van der Waals clashes with (**B**) A80 and G38 in FABP5 and (**C**) K58, N59, T60, E72, and T73 in FABP7. (**D**–**F**) Solvent exposure and van der Waals clashes of 15d-PGJ_2_ in (**D**) FABP3, (**E**) FABP5, and (**F**) FABP7.

Our studies revealed that 15d-PGJ_2_ exhibits highest affinity for FABP3 while binding to FABP5 and FABP7 is considerably lower (Table 1). Modeling analysis indicates that the ligand can be accommodated and stabilized in the binding pockets of all FABPs by engaging in H-bonding interactions (Fig. 5A–C). However, the rigidity of the cyclopentenone moiety conformationally constraints the ligand and exposes the tail of the acyl chain to the solvent (Fig. 5D–F), inducing van der Waals clashes in FABP5 and FABP7 to reduce binding affinity (Fig. 5A–C).

**Figure 6:**
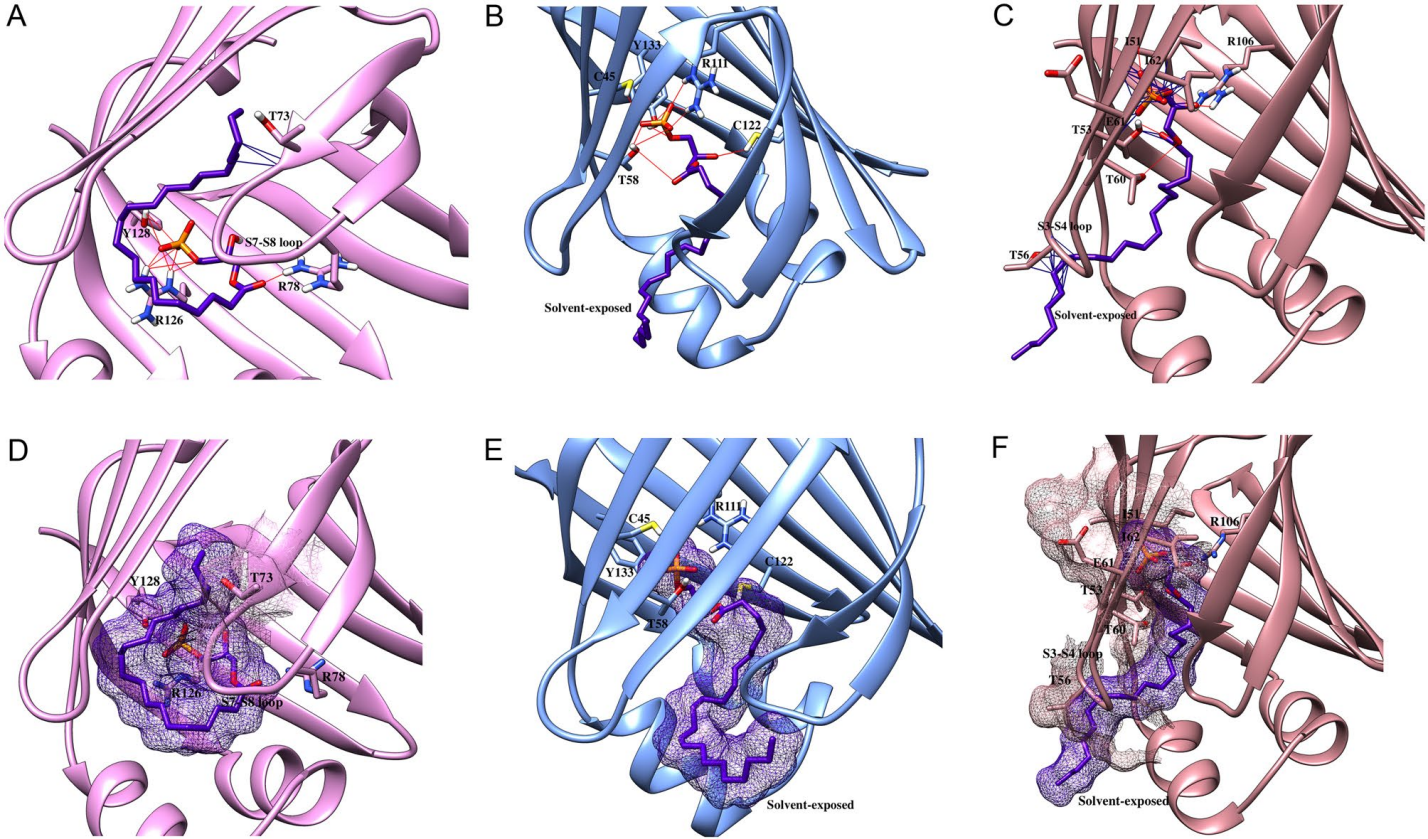
Docking poses LPA in FABP3, FABP5, and FABP7. LPA engages in numerous H-bonding interactions with (**A**) R126, Y128, and R78 in FABP3, (**B**) C45, T58, R111, C122, and Y133 in FABP5, and (**c**) I51, T53, T60, I62, and R106 in FABP7. (**D**–**F**) The orientation of LPA in (**D**) FABP3, (**E**) FABP5, and (**F**) FABP7. Note the solvent exposure in FABP5 and FABP7. The orientation of LPA also induces van der Waals clashes with (**D**) T73 in FABP3 and (**F**) I51, T53, T56, E61, and I62 in FABP7 to reduce binding affinity.

Similar to 15d-PGJ_2_, LPA displays highest affinity for FABP3 and lowest for FABP7 (Table 1). The phosphate group and ester moiety act as H-bond acceptors and engage in numerous interactions in the binding site of FABP3 (Fig. 6A), although this stabilization is partially offset by the placement of the acyl chain tail that leads to van der Waals clashes (Fig. 6A, 6D). Similar hydrogen binding interactions are observed in FABP5 (Fig. 6B). However, the wider pocket of FABP5 enhances solvent exposure of the acyl chain (Fig. 6E), which may counteract the stabilization obtained through hydrogen bonding to reduce affinity in FABP5. In the FABP7 binding pocket, the orientation of the phosphate and ester groups results in fewer hydrogen bonding interactions, leading to solvent exposure and substantial van der Waals clashes that compromise binding (Fig. 6C, F).

### sEH-mediated control of hippocampal glutamate synapses requires FABPs

**Figure 7:**
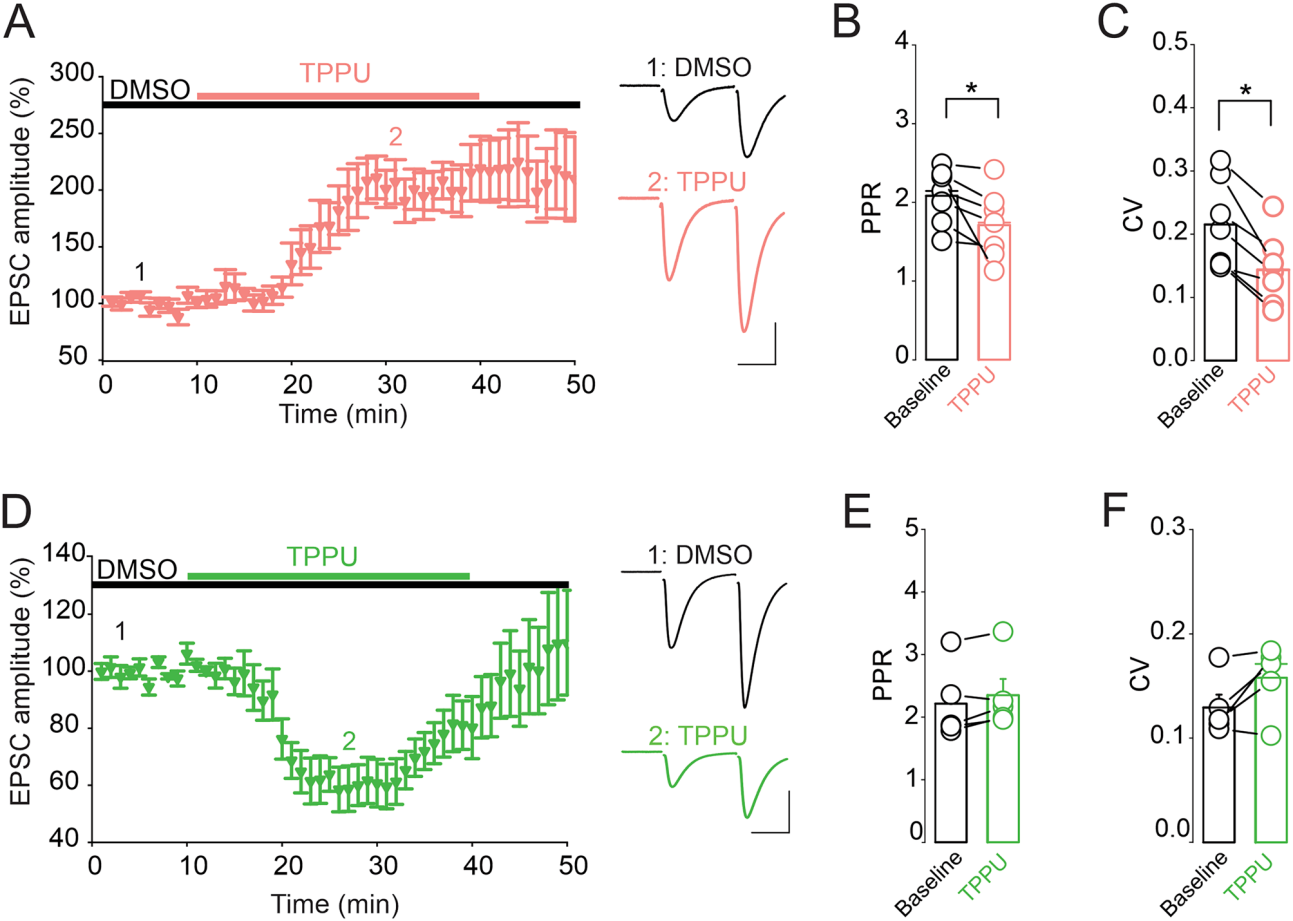
sEH inhibition bidirectionally gates glutamate synapses of CA1 pyramidal neurons. (**A**) Inhibition of sEH potentiates eEPSC amplitude in CA1 pyramidal neurons. Left graph is a summary of the time course and magnitude of the potentiation of EPSCs induced by TPPU (10 µM). Right panel depicts the average of at least thirty pairs of EPSCs collected at the time points indicated by the numbers in the left panel. Scale bars: 25 ms, 100 pA. (**B**) Averaged PPR of EPSCs obtained at baseline and during TPPU application. (**C**) Averaged CV of EPSCs obtained in absence and presence of TPPU. (**D**) Inhibition of sEH depresses eEPSC amplitude in CA1 pyramidal neurons. Left graph is a summary of the time course and magnitude of the depression of EPSCs induced by TPPU (10 µM). Right panel depicts the average of at least thirty pairs of EPSCs collected at the time points indicated by the numbers in the left panel. Scale bars: 25 ms, 100 pA. (**E**) Averaged PPR obtained in the absence and presence of TPPU. (**F**) Averaged CV of EPSCs obtained in absence and presence of TPPU.

The finding that EETs bind to FABPs with high affinity suggests the possibility that FABPs may regulate EET signaling in the brain. We first confirmed that inhibition of sEH, the primary hydrolase that degrades endogenous EETs, gates synaptic transmission in the hippocampal CA1 region as previously reported^23^. We found that the sEH inhibitor TPPU (10 µM) exerted bidirectional effects on the strength of CA1 glutamate synapses with a potentiation (Fig. 7A, n = 8 neurons, 5 mice) and depression (Fig. 7D, n = 7 neurons, 6 mice) of eEPSC amplitude observed in 47% and 41% of CA1 pyramidal neurons, respectively. The TPPU-induced potentiation of eEPSCs was associated with a significant decrease in both the paired pulse ratio (PPR) (Fig. 7B, n = 7, *p* = 0.042) and the coefficient of variation (CV) (Fig. 7C, n = 7, *p* = 0.0017), indicating an increase in the probability of glutamate release. In contrast, the depression of EPSCs was not accompanied with significant changes in both the PPR (Fig. 7E, n = 5, *p* = 0.108) and CV (Fig. 7F, n = 5, *p* = 0.086), thereby demonstrating that this effect was not mediated by changes in the probability of glutamate release. These results suggest that sEH metabolites, presumably EETs, bidirectionally gate the strength of CA1 pyramidal neuron glutamate synapses.

**Figure 8:**
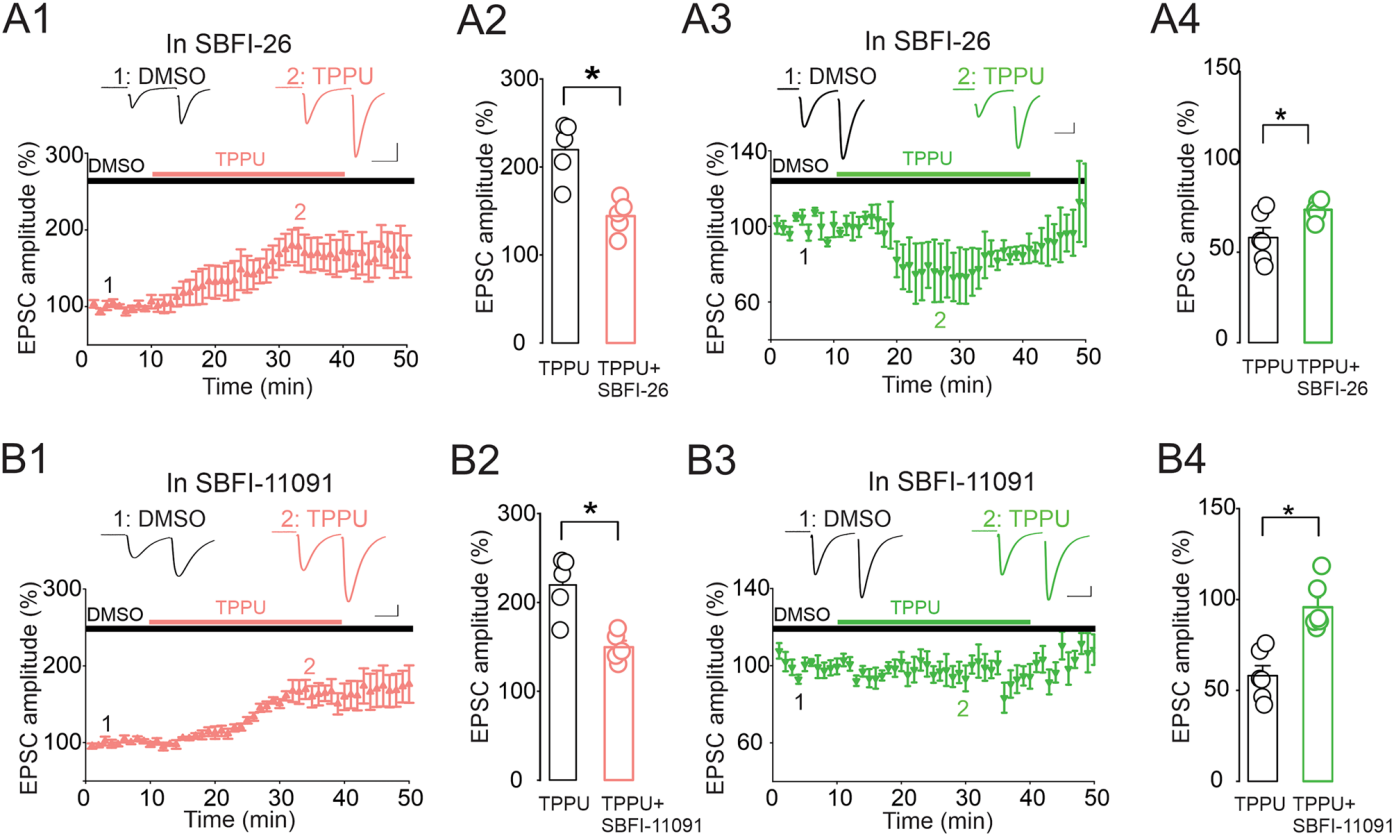
FABPs modulate sEH inhibition-mediated control of CA1 pyramidal neuron glutamate synapses. (**A**) The FABPs inhibitor SBFI-26 reduces the magnitude of TPPU-induced potentiation and depression of eEPSC amplitude. (**A1**) Lower panel depicts the magnitude of the eEPSC potentiation induced by TPPU (10 µM) in presence of SBFI-26 (10 µM). Upper graph is the average of eEPSC traces collected before (time 1) and during TPPU (time 2) administration. Scale bars: 25 ms, 100 pA. (**A2**) The average magnitude of TPPU-induced potentiation obtained in the absence and presence of SBFI-26. (**A3**) Lower panel depicts the magnitude of the eEPSC depression induced by TPPU (10 µM) in the presence of SBFI-26 (10 µM). Upper graph is a sample of averaged eEPSC traces collected before (time 1) and during TPPU (time 2) administration. Scale bars: 25 ms, 100 pA. (**A4**) The average magnitude of TPPU-induced depression obtained in the absence and presence of SBFI-26. (**B**) Potent FABP3 inhibitor reduces the magnitude of TPPU-induced potentiation of EPSCs. (**B1**) Summary of the TPPU-induced potentiation of eEPSCs obtained in the presence of SBFI-11091 (10 µM). Upper graph is a sample of eEPSC traces collected before (time 1) and during TPPU (time 2) administration. (**B2**) Averaged amplitude of eEPSCs obtained in TPPU and TPPU plus SBFI-11091. (B3–B4) Inhibition of FABP3 abolishes TPPU-induced depression of eEPSCs. (**B3**) Summary of the TPPU-induced depression of eEPSCs obtained in the presence of SBFI-11091 (10 µM). Upper graph is a sample of eEPSC traces collected before (time 1) and during TPPU (time 2) administration. Scale bars: 25 ms, 100 pA. (**B4**) Averaged eEPSCs amplitude obtained in TPPU and TPPU plus SBFI-11091. *p < 0.05.

We next examined the impact of FABP inhibition on the magnitude of TPPU-induced potentiation and depression of eEPSCs. Treatment of hippocampal slices with the FABP inhibitor SBFI-26 (10 µM) (Table 3) significantly reduced the TPPU-induced potentiation of eEPSCs (Fig. 8A1, A2, n = 5 neurons, 4 mice, *p* = 0.007; TPPU + SBFI-26 vs baseline, *p* = 0.00098; TPPU + SBFI-26 vs TPPU). Similar effects were observed on the TPPU-induced depression of eEPSCs (Fig. 8A3, A4, n = 6 neurons, 5 mice, *p* = 0.00041; TPPU + SBFI-26 vs baseline, *p* = 0.048; TPPU + SBFI-26 vs TPPU). As our results demonstrate that EETs display highest affinities for FABP3 and SBFI-26 is a weak FABP3 inhibitor (Table 3), we next assessed the impact of the potent FABP3 inhibitor SBFI-11091 (Table 3) on TPPU induced potentiation and depression of eEPSCs. We found that treatment of slices with SBFI-11091 (10 µM) reduced TPPU-induced potentiation of eEPSCs (Fig. 8B1, B2, n = 5 neurons, 4 mice, *p* = 0.0024; TPPU + SBFI-11091 vs baseline, *p* = 0.0015; TPPU + SBFI-11091 vs TPPU), an effect that was comparable in magnitude to that obtained with SBFI-26. In contrast, treatment with SBFI-11091 completely abolished TPPU-mediated depression of eEPSCs (Fig. 8B3, B4, n = 6 neurons, 5 mice, *p* = 0.469 TPPU + SBFI-11091 vs baseline, *p* = 0.00021; TPPU + SBFI-11091 vs TPPU). Taken together, these results position FABPs as novel modulators of sEH-dependent synaptic lipid signaling.

**Table 3:** Structures and FABP affinities of the inhibitors used in the current study.

Name	Structure	K_i_ FABP3 (μM)	K_i_ FABP5 (μM)	K_i_ FABP7 (μM)
SBFI-26		2.70 ± 0.42	0.81 ± 0.09	0.45 ± 0.07
SBFI-11091		0.21 ± 0.09	1.59 ± 0.24	1.36 ± 0.23

## Discussion

FABPs have recently emerged as key regulators of diverse neurological and behavioral processes including sleep, anxiety, reward, and pain. Although originally implicated in transporting medium and long chain fatty acids^1,2^, recent studies have expanded the repertoire of endogenous FABP ligands to include retinoic acid, eCBs, and LPA^35–37^. Our study demonstrates that EETs and 15d-PGJ_2_ are ligands of brain-expressed FABPs and implicates FABPs as modulators of synaptic EET signaling.

EETs are synthesized from AA by cytochrome P450 enzymes while sEH mediates their inactivation^38^. EETs activate distinct receptors and modulate neuronal excitability and synaptic function in the hippocampus^22–24^. Although the enzymatic machinery that controls EET levels has been identified, the mechanisms that regulate EET transport have remained undefined. Here, we expand upon previous findings that EETs interact with FABP3^25^ by further demonstrating that EETs are high affinity ligands for FABP3 and FABP5. Such results suggest that these FABPs may gate EET function in the brain. Indeed, previous studies have shown that 14,15-EET potentiates while 11,12-EET depresses hippocampal glutamate synapses^22,23^. Consistent with these observations, our functional studies demonstrate that the bidirectional control of hippocampal glutamate synapses induced by sEH inhibition requires FABPs. Molecular docking highlighted major contributions of van der Waals clashes and solvent exposure as determinants underlying the differential affinities between EETs and FABPs and supported our binding studies revealing that EETs display highest affinities for FABP3. Consistent with these findings, a potent FABP3 inhibitor abolished TPPU-induced synaptic depression in the hippocampus, suggesting that this protein may be necessary for EET signaling. Our recent work demonstrating that FABP5 serves an obligate role in regulating synaptic eCB transport^7,9^ coupled with the current findings that FABP3 may modulate EET (and potentially other sEH metabolite) function, suggests that different FABP subtypes may control the signaling of distinct lipid messengers.

The present findings indicate that sEH inhibition potentiates and depresses glutamate synapses by presynaptic and postsynaptic mechanisms, respectively. These opposing effects may involve recruitment of different EET species and their downstream signaling cascades, and/or non-EET cytochrome P450 or sEH metabolites. For example, cytochrome P450 metabolites of AEA, docosahexanoyl ethanolamide, and eicosapentaenoyl ethanolamide function as potent CB2 agonists^39,40^, and it is conceivable that they may engage additional targets that modulate synaptic function. Furthermore, it is possible that epoxydocosapentaenoic and epoxyeicosatetraenoic acids as well as their bioactive vicinal diol metabolites may also regulate synaptic signaling^41^. However, our functional studies are consistent with recent work demonstrating that 14,15-EET and 11,12-EET mediate potentiation and depression of hippocampal glutamate synapses, respectively^22,23^. Our finding that 11,12-EET exhibits high affinity for FABP3 combined with the observation that TPPU-induced synaptic depression is completely blocked by the potent FABP3 inhibitor SBFI-11091 suggests that 11,12-EET transport by FABP3 may underlie the depression of glutamate synapses in CA1 pyramidal neurons. Interestingly, while 14,15-EET likewise displays high affinity for FABP3, SBFI-11091 was unable to fully blunt the potentiation of EPSCs indicating that FABP3 may exert a partial role in mediating 14,15-EET signaling. Alternatively, despite its weak affinity for FABP5 and FABP7, these proteins may also contribute to 14,15-EET transport.

The suggestion that endogenous EETs regulate synaptic transmission in the CA1 hippocampal region is consistent with the expression of the EET biosynthetic enzyme family CYP2J in pyramidal neurons^22^ and the distribution of sEH to astrocytes and neurons^42–44^. Furthermore, the exclusive localization of FABP3 to neurons^3^ positions this protein as a likely regulator of neuronal hippocampal EET signaling. Although the mechanism(s) by which FABPs control EET signaling remains unknown, the lipophilicity of EETs likely necessitates intracellular and/or extracellular transport by FABPs to their respective receptors as observed with eCBs^7,16,45^. Moreover, as FABP5 and FABP7 are expressed in astrocytes, it is tempting to speculate that they may contribute to the trafficking and metabolism of astrocytic EETs. It is noteworthy that low levels of endogenous EETs are observed in the brain and our study did not quantify the impact of sEH or FABP inhibition upon EET concentrations^18^. Future studies will be required to define the precise contribution of each FABP isoform in modulating EET levels and function in vivo.

Our results additionally extend previous work by demonstrating that FABPs accommodate subsets of eicosanoids. While 15-Hydroxyeicosatetraenoic acid and leukotriene A4 bind to FABP5, PGE_2_ lacks affinity for FABP5 and FABP7^14,15^. We expanded upon these findings by demonstrating that PGE_2_ and PGF_2α_ do not display appreciable affinities for any of the brain expressed FABPs and similar results were observed for PGE_2_-GE and PGF_2α_-EA, the cyclooxygenase-2 metabolites of eCBs. Interestingly, the structurally related PPARγ agonist 15d-PGJ_2_ exhibited considerable affinity for FABP3 (Fig. 2, Table 1), suggesting that FABP3 may play a key role in regulating its nuclear translocation. LPA emerged as a ligand for FABP3 and FABP5 while weak interactions with FABP7 were noted. Molecular modeling indicated that van der Waals clashes and exposure of the acyl chain to bulk solvent likely contribute to the poor affinities of 15d-PGJ_2_ and LPA for FABP7. In addition to PPARγ, LPA is a canonical agonist at cell surface LPA receptors^46^, highlighting the possibility that FABP3 and/or FABP5 may regulate distinct aspects of LPA transport including its nuclear transport to PPARγ. Collectively, our study identifies novel lipid ligands for FABPs and uncovers a key function for this protein family in controlling the function of sEH metabolites at central synapses.

## Methods

### Compounds

5,6-EET (#50211), 8,9-EET (#50351), 11,12-EET (#50511), 14,15-EET (#50651), PGE_2_-GE (#10140), PGE_2_ (#14010), PGF_2α_ (#16010), PGF_2α_-EA (#16013), 15d-PGJ_2_ (#18570), LPA (#10010093), and 11-(dansylamino) undecanoic acid (DAUDA, #10005188) were from Cayman Chemical. 8-anilino-1-naphthalenesulfonic acid (ANS, #A-1028) was from Sigma. SBFI-26^17^ and SBFI-11091 (referred to previously as α-19^31^) were synthesized as described^17,31^.

### Ethics statement

All experimental procedures were approved by the University at Buffalo Animal Care and Use Committee (#RIA01023N). Mice were housed in an AAALAC certified facility with ad libitum access to food and water, and lighting was maintained on a 12-h light/dark cycle. The electrophysiological experiments were performed on 8- to 12-week old male C57Bl/6 mice. The experiments were performed in accordance with recommendations in the ARRIVE guidelines and the American Veterinary Medical Association guidelines for the euthanasia of animals.

### FABP binding studies

Affinities of each ligand were determined via fluorescent probe displacement from purified FABPs as previously described^30,31,47^. Ligands were incubated in assay buffer (30 mM Tris, 100 mM NaCl, pH 7.5) containing 3 μM purified FABPs, 500 nM DAUDA (for FABP3 and FABP5) or ANS (for FABP7). AA (10 μM) was included to account for maximal probe displacement. The fluorescence signal was quantified on a F5 Filtermax Multi-Mode Microplate Reader (Molecular Devices) with the excitation and emission wavelengths set at 360/535 nm for DAUDA and 360/465 nm for ANS. After background subtraction, normalized fluorescence values were fit to a one-site binding model using GraphPad Prism (v 9.5) and Ki values were determined using the following equation: Ki = IC_50_/(1 + ([Probe]/Kd).

### Molecular docking

The docking study of the FABPs was carried out using DOCK6.9^48^ by following a four-step protocol of preparing the FABP receptors for docking, creating the 3D molecular formats of the ligands, docking analysis, and evaluating the predicted binding affinities of the ligands. (i) As the ligands used in the study were based on AA, and there are no co-crystal structures of FABP with AA, co-crystal structures of FABP with C18 fatty acids (PDB codes: 1HMS for FABP3, 4LKT for FABP5, and 1FE3 for FABP7) were used as they closely resemble the AA. Proteins in the co-crystal structures were cleaned by removing small molecules and processed through the Dock Prep module of UCSF Chimera to add hydrogens and partial atom charges under the AMBER FF14SB^49^ protein parameters. Docking spheres were generated over the surface contours of the proteins and those within 10 Å of the fatty acid reference ligands were retained for docking anchor placement. The docking grid was calculated using a box with an 8 Å cutoff from the reference ligands with a grid point spacing of 0.4 Å. The electrostatic interactions were modeled with a distance-dependent dielectric of 4r and a 6–9 Lennard–Jones potential for van der Waals interactions. (ii) The SMILE strings of the AA-based ligands were downloaded from Cayman Chemicals Catalog (www.caymanchem.com) and were converted to 2D molecular structures via PerkinElmer’s ChemDraw and saved in the MOL format. These coordinates were parsed through the Avogadro^50^ molecular editor to generate 3D structures in the biologically relevant protonation state (at pH 7.4) using parameters of the integrated Open Babel^51^ toolkit. The internal energy minimization was done by the Merck Molecular Force Field (MMFF94) until the energy gradient reached approximately 0.0 kJ/mol, and then the coordinates were written in the MOL2 format. (iii) Docking calculations of prepared MOL2 files were carried out using flexible (FLX) and fixed anchor docking algorithm (FAD) docking protocols in DOCK6.9 by keeping the carbonyl of the carboxylate moiety as the anchor. (iv) Finally, the representative docking solution was chosen compared to the docked AA reference based on the structural overlap and the RMSD (less than 2 Å). The visualization and depiction of selected docking results were accomplished through UCSF Chimera molecular visualization programs.

### Acute brain slice preparation

Mice were deeply anesthetized with isoflurane and decapitated. Coronal brain slices (350 μm thick) containing the hippocampus were cut using a vibratome Leica VT 1200 (Leica, CA) in a modified ice-cold chlorine-based artificial cerebrospinal fluid (ACSF) composed in mM: 110 mM choline-Cl, 2.5 mM KCl, 0.5 mM CaCl_2_, 7 mM MgSO_4_, 1.25 mM NaH_2_PO_4_, 26.2 mM NaHCO_3_, 11.6 mM sodium l-ascorbate, 3.1 mM sodium pyruvate, and 25 mM glucose, and equilibrated with 95% O_2_ and 5% CO_2_. Hippocampal slices were first transferred to a chamber with the same cutting solution at 35 °C for 15 min and then to a standard ACSF in mM: 119 mM NaCl, 2.5 mM CaCl_2_, 1.3 mM MgSO_4_, 1 mM NaH_2_PO_4_, 26.2 mM NaHCO_3_, and 11 mM glucose continuously bubbled with a mixture of 95% O_2_ and 5% CO_2_ for additional 45 min at 35 °C. Slices were allowed to recover at room temperature (≥ 1 h). After recovery, individual slice was transferred to a recording chamber, continuously perfused with standard ACSF containing 0.01% DMSO saturated with 95% O_2_ and 5% CO_2_, and warmed at 30 °C.

### Ex-vivo whole-cell recordings

Hippocampal CA1 pyramidal neurons were visualized using an upright microscope (BX 51 WI; Olympus, Tokyo, Japan) and somatic whole-cell recordings were obtained from pyramidal neurons using glass pipette electrodes with 3–5 MΩ resistance when filled with an internal solution containing: 120 mM potassium gluconate, 10 mM KCl, 10 mM Na_2_-phosphocreatine, 10 mM HEPES, 1 mM MgCl_2_, 1 mM EGTA, 2 mM Na_2_-ATP, 0.25 mM Na-GTP (pH 7.3 adjusted with KOH; osmolarity, 280–290 mOsmol/l). All recordings were performed in the presence of the GABA A receptor antagonist picrotoxin (100 μM). Excitatory postsynaptic currents (EPSCs) were evoked with electrical pulses (5–20 V, 100–200 μs) delivered at 0.1 Hz using glass microelectrode placed in the striatum radiatum in CA1 pyramidal neurons voltage-clamped at − 70 mV. All recorded currents were amplified with an a Multiclamp 700B amplifier (Molecular Devices, Sunnyvale, CA), filtered at 3 kHz, digitized at 20 kHz with Digidata 1200 (Molecular Devices), and acquired using the pClamp 10.7 software (Molecular Devices, Sunnyvale, CA). To examine the impact of TPPU on the amplitude of evoked EPSCs, at least 10–20 min of stable baseline recordings were obtained and TPPU was perfused to the brain slices (final DMSO concentration of 0.01%).

### Data analysis

For the analysis of the electrophysiology data, the amplitude of evoked EPSCs (eEPSC) was determined by measuring the average current during a 2-ms period at the peak of each eEPSC and subtracted from the average baseline current determined during a 5-ms window taken before the stimulus artifact. All eEPSC amplitudes were normalized to the mean baseline amplitude recorded for at least 10 min before drug application. All the data are represented as the mean ± SE and were not transformed. The normal distribution of the data was tested using the Shapiro–Wilk test and all the data exhibited normal distribution, statistical analyses were therefore conducted using the paired and unpaired t-test for comparison within and between groups, respectively.

### Supplementary Information

Supplementary Figures.

## Data Availability

Data will be available from the corresponding author upon reasonable request.
