# Supplementary material for: Fatty acid binding proteins are novel modulators of synaptic epoxyeicosatrienoic acid signaling in the brain
Source: Sci Rep. 2023 Sep 14;13:15234. doi: 10.1038/s41598-023-42504-4 (PMC10502087; doi:10.1038/s41598-023-42504-4)
Supplement: Supplementary file 1 — Supplementary Figures. [file 41598_2023_42504_MOESM1_ESM.docx]

**
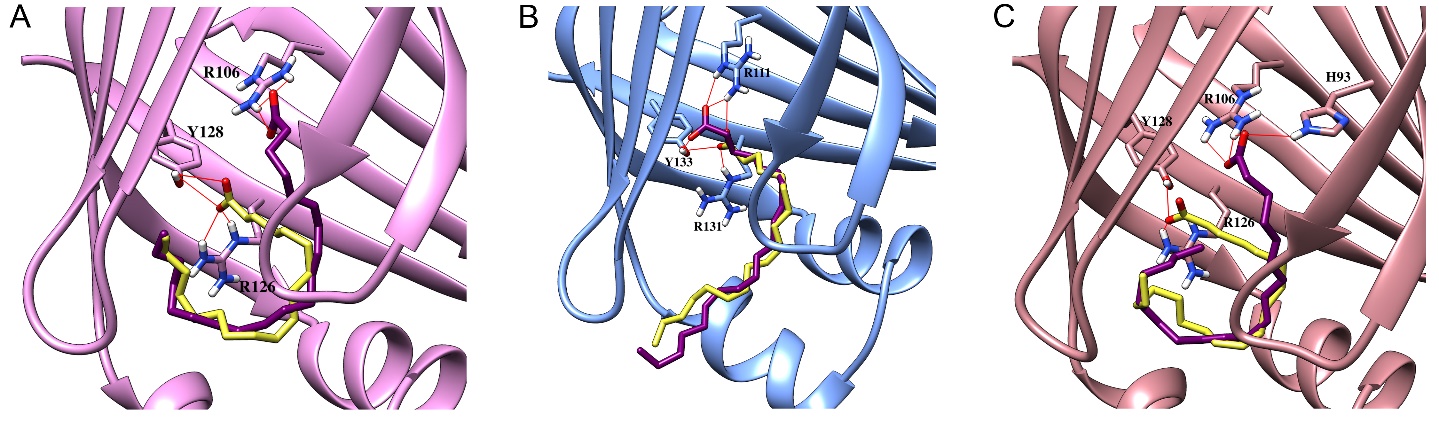
**

**Supplementary Figure 1.** Docking poses and interactions of AA in FABP3, FABP5, and FABP7. (**a-c**) Docking of AA (magenta) was based upon the co-crystal structures of (**a**) oleic acid in FABP3, (**b**) linoleic acid in FABP5, and (**c**) oleic acid in FABP7. AA was docked into the FABPs and used as the structural basis for binding pose analysis. Due to its increased chain length, AA orients the carboxylate moiety in conformations distinct from the parent ligands.

**
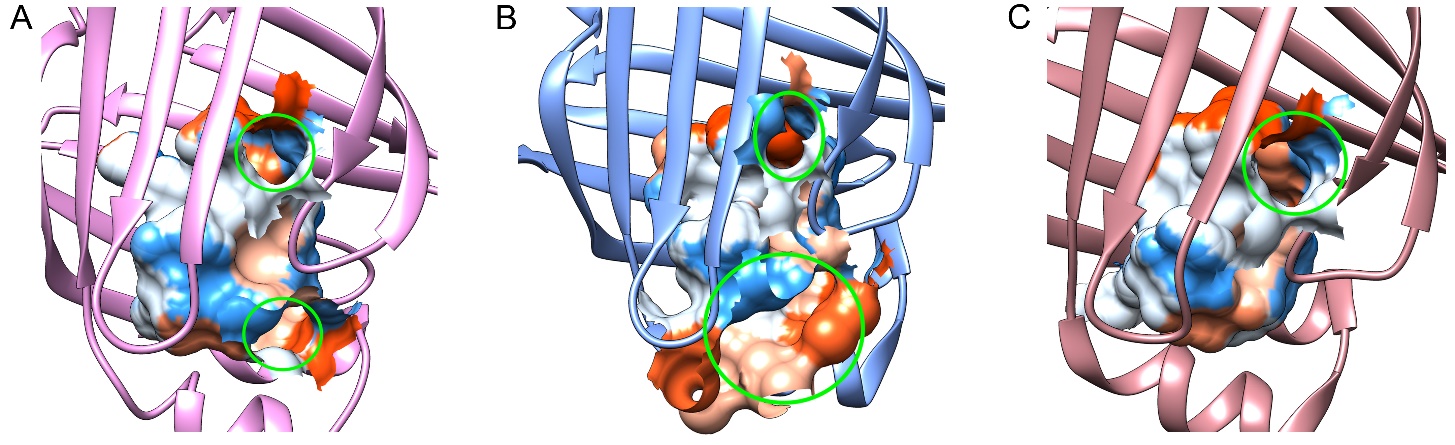
**

**Supplementary Figure 2.** Volumes and surface areas of the FABP3, FABP5, and FABP7 binding pockets. Respective binding pocket volumes and surface areas represented as electrostatic surfaces based on the solvent-accessible surface of (**a**) FABP3 (303.614 Å3, 423.846 Å2), (**b**) FABP5 (332.770 Å3, 491.063 Å2), and (**c**) FABP7 (323.333 Å3, 379.124 Å2). FABP3 and FABP7 have more compact binding pockets compared to FABP5, while the FABP5 binding pocket contains a wider entrance compared to FABP3 and FABP7. Calculations were performed using CATSp (http://sts.bioe.uic.edu/castp/index.html?2r7g).

.


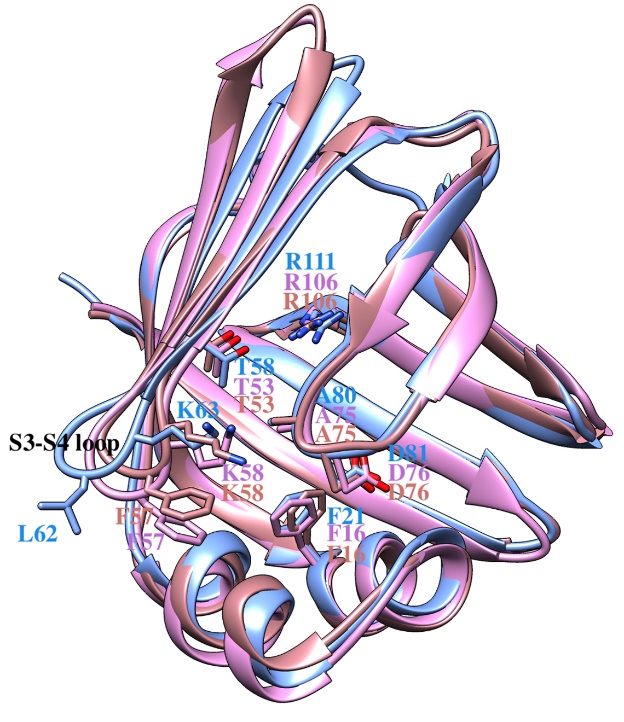


**Supplementary Figure 3.** Overlay of the FABP3, FABP5, and FABP7 structures. Overlay of FABP3 (pink), FABP5 (blue), and FABP7 (beige) shows differences in binding site residues. In FABP3 and FABP7, F57 in the S3-S4 loop attains an inward-directed orientation toward the binding pocket while in FABP5, L62 is outward facing.
